# Dreaded Degos Disease in Childhood

**DOI:** 10.7759/cureus.76844

**Published:** 2025-01-03

**Authors:** Murugan Sundaram, Nishantan Thamotharan, Adikrishnan Swaminathan, Sudha Rangarajan, Krishnakanth Muralidhar

**Affiliations:** 1 Dermatology, Sri Ramachandra Institute of Higher Education and Research, Chennai, IND

**Keywords:** cutaneous ulcer, degos disease, female child, malignant atrophic papulosis, porcelain white scars

## Abstract

Degos disease or malignant atrophic papulosis is a rare multisystem vascular disease of unknown etiology most commonly affecting the small- to medium-sized arteries of the dermis of the skin, gastrointestinal tract, and central nervous system. Endothelial cell damage and the activation of the coagulation cascade leading to the obliterative thrombosis of the vessel wall are the main pathogenic mechanisms behind the disease. The characteristic cutaneous features are porcelain white atrophic papules with a peripheral rim of erythema seen commonly over the trunk, chest, and upper and lower limbs. In Degos disease, cutaneous and systemic manifestations typically do not present simultaneously, with skin lesions often appearing first. Systemic involvement, affecting organs such as the gastrointestinal tract or central nervous system, may develop months or years later. Patients should be closely monitored for potential systemic complications, as these significantly impact prognosis, necessitating multidisciplinary care to ensure early detection and management. The disease carries a poor prognosis because of systemic involvement. Degos disease is extremely rare in children with very few cases being reported in literature. We hereby report a rare case of Degos disease in a five-year-old female child with lesions confined to the skin with a lack of systemic involvement.

## Introduction

The term Degos disease was initially described by Köhlmeier in the year 1941 [1]. Degos later described the disease as a separate entity and named the disease [2]. The exact incidence of Degos disease is unknown, and no specific genetic mutations or etiologies have been identified in the literature. It is considered a rare disorder with an unclear pathogenesis, thought to be a form of vasculopathy leading to the thrombotic occlusion of small- and medium-sized vessels. The condition exhibits a marked male predominance, with a documented male-to-female ratio of approximately 3:1 [3]. The disease when restricted to the skin is known as benign atrophic papulosis, and when systems other than the skin like the gastrointestinal and central nervous system (CNS) are involved, it is called malignant atrophic papulosis. The severity of Degos disease depends upon systemic involvement, not by malignancy of cells or tissues. The pediatric population is rarely affected by the disease. There have been limited reports of cases in pediatric populations, with evidence suggesting that multisystem involvement is more prevalent in children, particularly with a male predominance, compared to adults [4]. In this report, we describe a rare case of a five-year-old child diagnosed with benign cutaneous Degos disease, characterized by the absence of visceral involvement. To date, approximately 34 cases of Degos disease have been reported in the pediatric age group, making this case potentially the 35th documented instance.

## Case presentation

**Figure 1:**
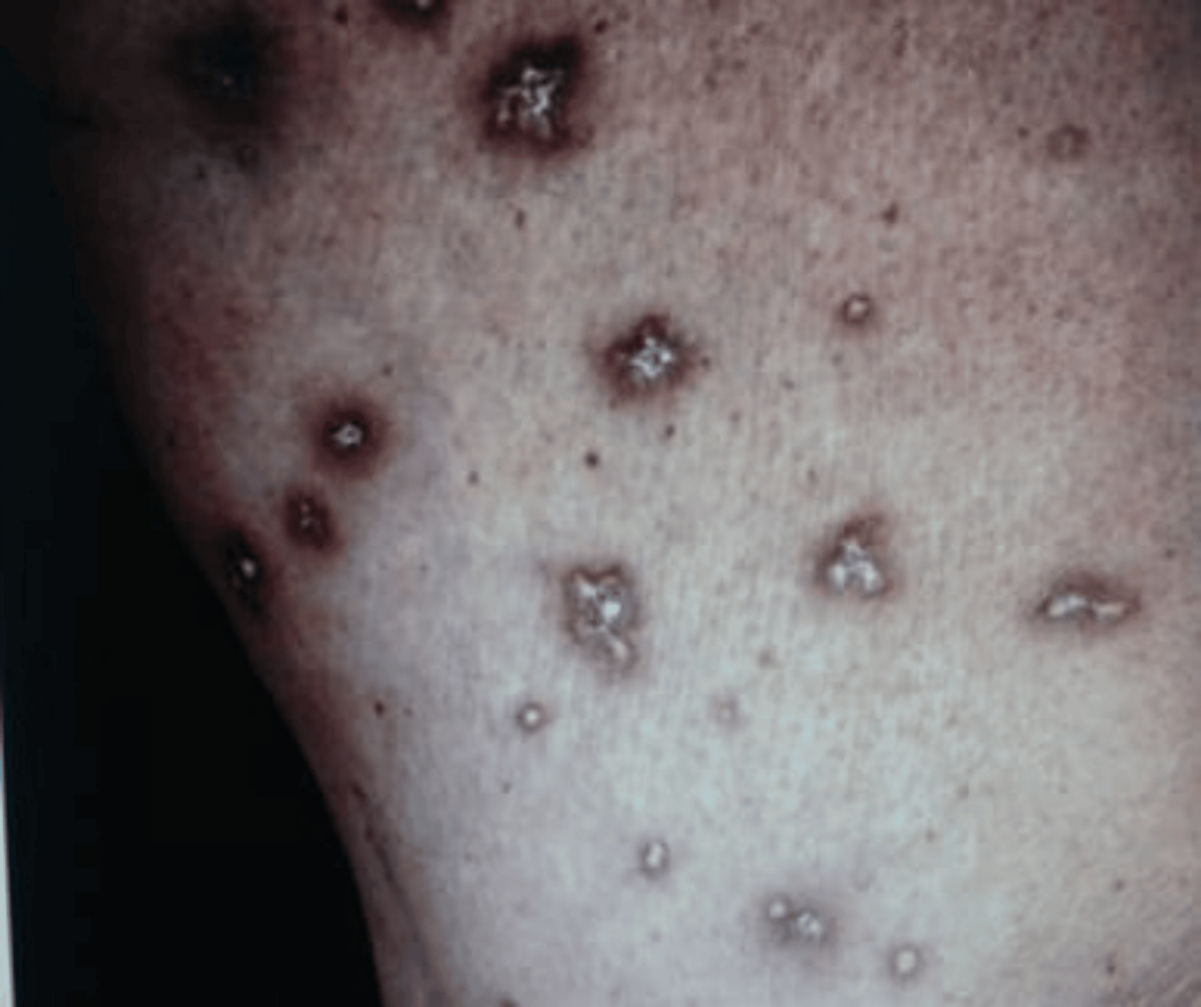
Multiple ulcers with central hemorrhagic crusting over the trunk

A female child, aged five years, presented with complaints of painful ulcers over the upper back, lower abdomen, and arms for the past six months. The cutaneous lesions presented as multiple ulcers of size ranging from 2×2 cm to 2×4 cm, with central hemorrhagic crusting observed on the arms, chest, lower abdomen, and back. These lesions began as red papules that gradually enlarged, progressed into ulcers, and eventually healed, leaving behind porcelain white atrophic scars (Figure 1). 

There was no history of fever, vomiting, abdominal/joint pain, diarrhea, melena, and drug intake in the child. Also, there was no family history of Degos disease and autoimmune diseases. The child was initially treated for impetigo by a pediatrician with povidone-iodine and antibiotics providing no relief. General examination and systemic examination revealed no abnormalities.

**Figure 2:**
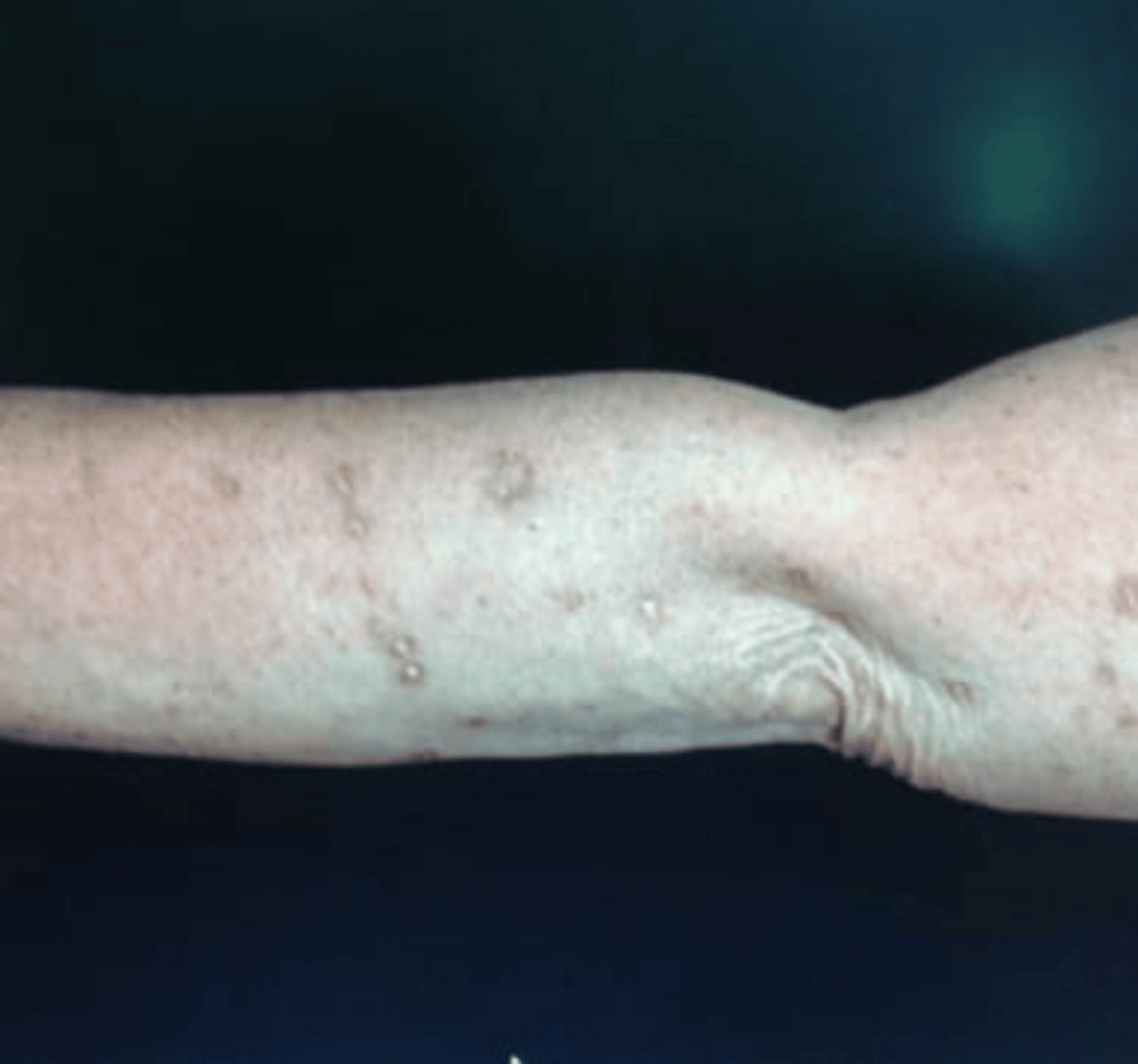
Porcelain white scars over the forearm

The palms and soles were not involved. Porcelain white atrophic scars were seen in healed lesions over the forearms (Figure 2). The hair, nails, and mucosa were normal.

**Figure 3:**
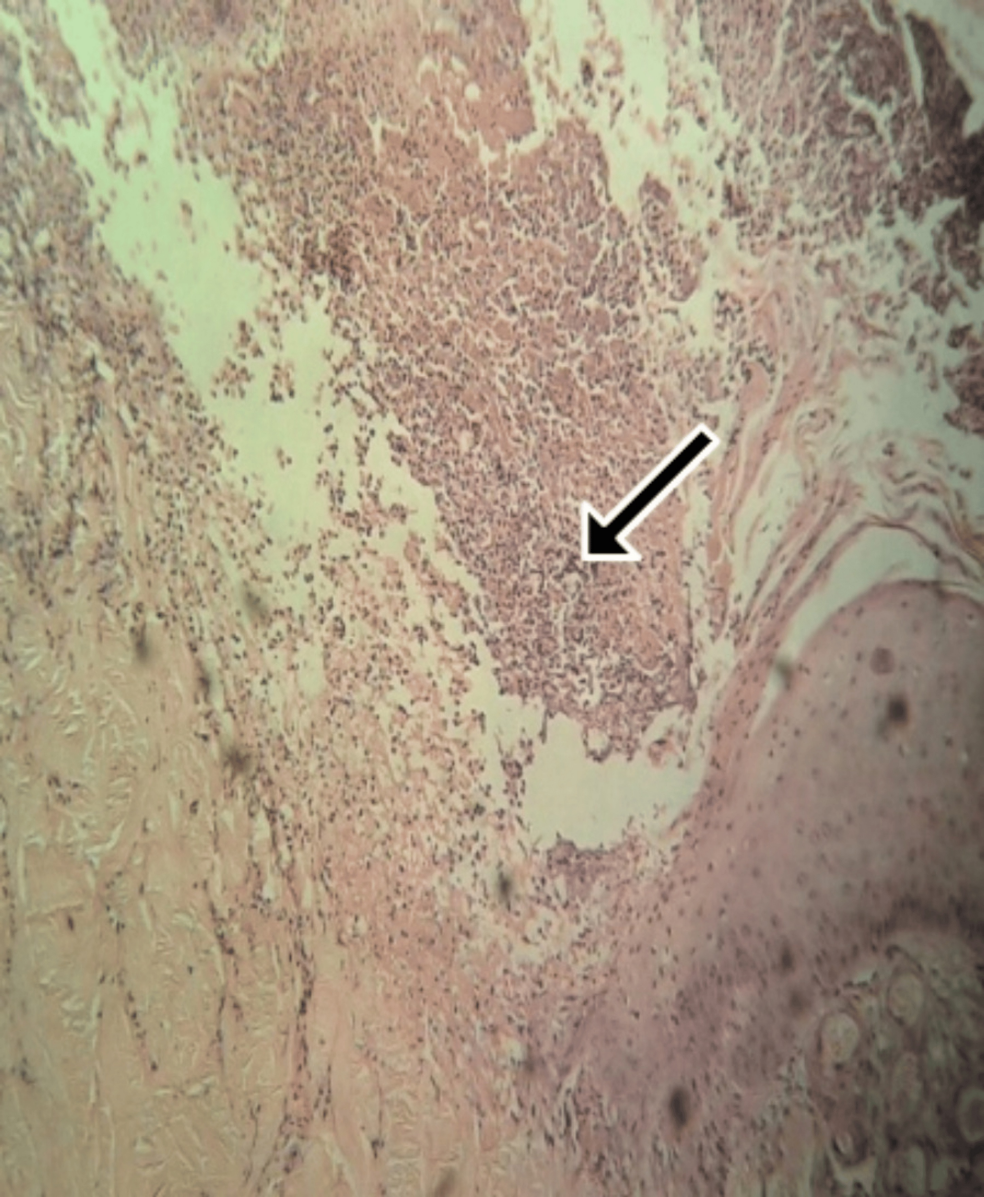
Hematoxylin and eosin staining under 40× magnification with the arrow pointing towards a wedge-shaped area of infract in the dermis

Differential diagnoses of erythema multiforme, papulonecrotic tuberculid, and lymphomatoid papulosis were considered. In the early stages, the histopathological differential diagnosis includes cutaneous lupus, as its presentation may resemble the initial lesions seen in this condition. Skin biopsy was done, and histopathology showed a wedge-shaped area of infarct in the dermis with lymphocytic infiltrate consistent with Degos disease (Figure 3). Laboratory studies were normal. Abdominal USG, abdominal CT, brain CT, and lower gastrointestinal endoscopy were normal. Immunohistochemistry (IHC) was not performed in this child.

The child was diagnosed to have benign cutaneous Degos disease with the absence of gastrointestinal and CNS involvement. She was managed symptomatically with topical zinc and antibacterial creams and was referred to a pediatrician for further evaluation and management. However, the patient was lost to follow-up during the subsequent course of the condition.

## Discussion

Degos disease is a thrombo-occlusive disorder of unknown etiology [5], predominantly affecting blood vessels of the skin. The complement complex C5b-9, also known as the membrane attack complex, and the upregulation of type 1 interferon-inducible protein have been lately identified to play a main role in the pathogenesis of the disease [6]. Most of the cases are sporadic affecting males commonly, but familial variants have also been reported [7].

Clinically, it starts initially as multiple rose-colored papules over the trunk and extremities that later become umbilicated with the development of ulcers, and finally, a central porcelain white region of atrophy surrounded by an erythematous rim is formed [8]. Systemic involvement determines the disease prognosis. The gastrointestinal system and CNS are most commonly involved [9]. Gastrointestinal symptoms are hematemesis, abdominal pain, and dyspepsia. Bowel infraction and peritonitis are common causes of mortality in Degos disease [10]. CNS involvement presents as hemorrhagic or ischemic stroke and polyradiculoneuropathy [11].

The typical histopathological feature of the disease is an inverted wedge-shaped area of central necrosis with sparse lymphocytic infiltrate in the upper dermis [12]. The differential diagnosis of the disease includes primary antiphospholipid syndrome or antiphospholipid syndrome caused by systemic lupus erythematosus or other connective tissue diseases [2].

Treatment of Degos disease is mainly with antiplatelet drugs like aspirin [13], dipyridamole, and pentoxifylline. Heparin and other fibrinolytics also provide good benefits. Other options are warfarin, dextrans, chloroquine, immunosuppressives like methotrexate, and plasma exchange. Recently, eculizumab, a terminal complement inhibitor, and treprostinil, a prostacyclin analog, have been found to be effective in treating the disease [14].

Ng and Koh [15] reported a unique case of Degos disease in a child with acute renal failure. Shi et al. [16] reported an instance of infant-onset Degos disease with CNS involvement. Wang et al. [17] reported a case of pediatric malignant atrophic papulosis with positivity for anticardiolipin antibodies and small bowel perforation.

## Conclusions

Degos disease is a rare disease with diagnostic and therapeutic challenges. Early diagnosis and frequent screening for systemic organ involvement are very essential to identify complications and to improve the prognosis of the disease. This case carries a better prognosis as the child had a disease limited only to the skin with the absence of systemic involvement but requires frequent surveillance as they may develop systemic features at a later date.
